# The Effect of Swedish Snuff (Snus) on Offspring Birthweight: A Sibling Analysis

**DOI:** 10.1371/journal.pone.0065611

**Published:** 2013-06-12

**Authors:** Sol Pía Juárez, Juan Merlo

**Affiliations:** 1 Centre for Economic Demography, Lund University, Lund, Sweden; 2 Unit for Social Epidemiology, Department of Clinical Sciences, Faculty of Medicine, Lund University, Lund, Sweden; University of Cincinnati, United States of America

## Abstract

Current observational evidence indicates that maternal smoking during pregnancy is associated with reduced birthweight in offspring. However, less is known about the effect of smokeless tobacco on birthweight and about the possible mechanisms involved in this relationship. This paper studies the effect of Swedish smokeless tobacco (snus) on offspring birthweight comparing the results obtained from a conventional linear regression analysis and from a quasi-experimental sibling design using a multilevel linear regression analysis. From the Swedish Medical Birth Register, we investigated 604,804 singletons born between 2002 and 2010. From them, we isolated 8,861 siblings from 4,104 mothers with discrepant snus-use habits (i.e., women who had at least one pregnancy during which they used snus and at least one other pregnancy in which they did not). The conventional analysis shows that continuous snus use throughout the pregnancy reduces birthweight in 47 g while quitting or relapsing snus has a minor and statistically non-significant effect (−6 g and −4 g, respectively). However, using a sibling analysis the effect observed for mothers who continue to use snus during pregnancy is less intense than that observed with previous conventional analyses (−20 g), and this effect is not statistically significant. Sibling analysis shows that quitting or relapsing snus use after the first trimester slightly reduces birthweight (14 g).However, this small change is not statistically significant. The sibling analysis provides strong causal evidence indicating that exposure to snus during pregnancy has a minor effect on birthweight reduction. Our findings provide a new piece of causal evidence concerning the effect of tobacco on birthweight and support the hypothesis that the harmful effect of smoking on birthweight is not mainly due to nicotine.

## Introduction

Maternal smoking during pregnancy is considered the most important preventable risk factor on offspring birthweight reduction [1]–[7]. Although the specific underlying mechanism is not yet well established, most studies agree that cigarette smoke has toxics such as carbon monoxide (CO), nicotine, metals and thousands of constituents of unknown toxicity [4]. In spite of this general consensus, nicotine remains the most widely discussed mechanism. Some evidence suggests that nicotine impairs tissue oxygenation and thereby placental function, resulting in fetal hypoxia and malnutrition which can lead to birthweight reduction, among other adverse effects [7]–[9]. Nevertheless, other studies have shown that the offspring of passively and actively smoking mothers experience a similar birthweight reduction [10]–[13], which suggests that the association between smoking during pregnancy and birthweight may be mediated by toxic products from tobacco combustion rather than by nicotine or by unmeasured familial confounding. In this line, a previous animal study showed that carbon monoxide is responsible for the reduction of fetal weight in rats, while nicotine linked to a reduction of the mother’s weight gain during pregnancy [14]. However, these findings need to be confirmed in human studies because humans may inhale smoke and metabolize toxins in a different way than in the controlled experimental environment of animal studies [7], [15]. In human observational studies it is shown that products of nicotine replacement therapy do not affect birthweight reduction [16]. However, this evidence may be difficult to extrapolate, since women who are willing to quit tobacco may not be representative of the entire population. This further overlooks the fact that smoking is not evenly distributed throughout the population and, therefore, mothers who quit smoking during pregnancy probably differ from those who continue smoking.

Our aim is to study the effect of smokeless tobacco (Swedish “snus”) during pregnancy on offspring birthweight. Swedish snus differs from other types of snuff products as it contains lower levels of harmful substances [17], [18]. It is composed of ground tobacco mixed with water, salt, sodium bicarbonate, sodium chloride, humectants and flavoring [17]. Snus cannot be classified either as a pure nicotine product, because it contains flavoring (less than 1%), or as a product of nicotine replacement therapy, because it has twice as much nicotine concentration [17]. However, snus provides a unique opportunity to eliminate the effect of combustion and, to some extent, isolate the effect of nicotine (with similar levels to those of smoked tobacco) on birthweight in an observational representative study.

A Swedish study showed that snus is associated to birthweight reduction [19]. However, this association may be confounded by familial factors linked with both snus use and birthweight. Therefore, we confront the results obtained with a conventional analysis (e.g., linear regression model) with a quasi-experimental sibling design which has been found to provide stronger causal evidence [20]–[22] by studying, ceteris paribus, the effect of differential exposure (snus use vs. non-snus use) on individuals who share the same genetic and social background [23], [24]. Therefore, this paper contributes not only to a better understanding of the mechanisms through which smoking reduces birthweight, but also to the debate on the health implications of using snus [25]–[27].

## Materials and Methods

### Ethics Statement and Study Population

The database used in our study was constructed by the National Board of Health and Welfare in coordination with Statistics Sweden, and it was approved by the Regional Ethical Review Board in Southern Sweden. Lund University signed a contract of confidentiality with Swedish Authorities; however, in the data we analyzed, the identification numbers were replaced with arbitrary numbers to safeguard the anonymity of the subjects. Active informed consent was waived as a requirement for the construction of the database.

**Figure 1:**
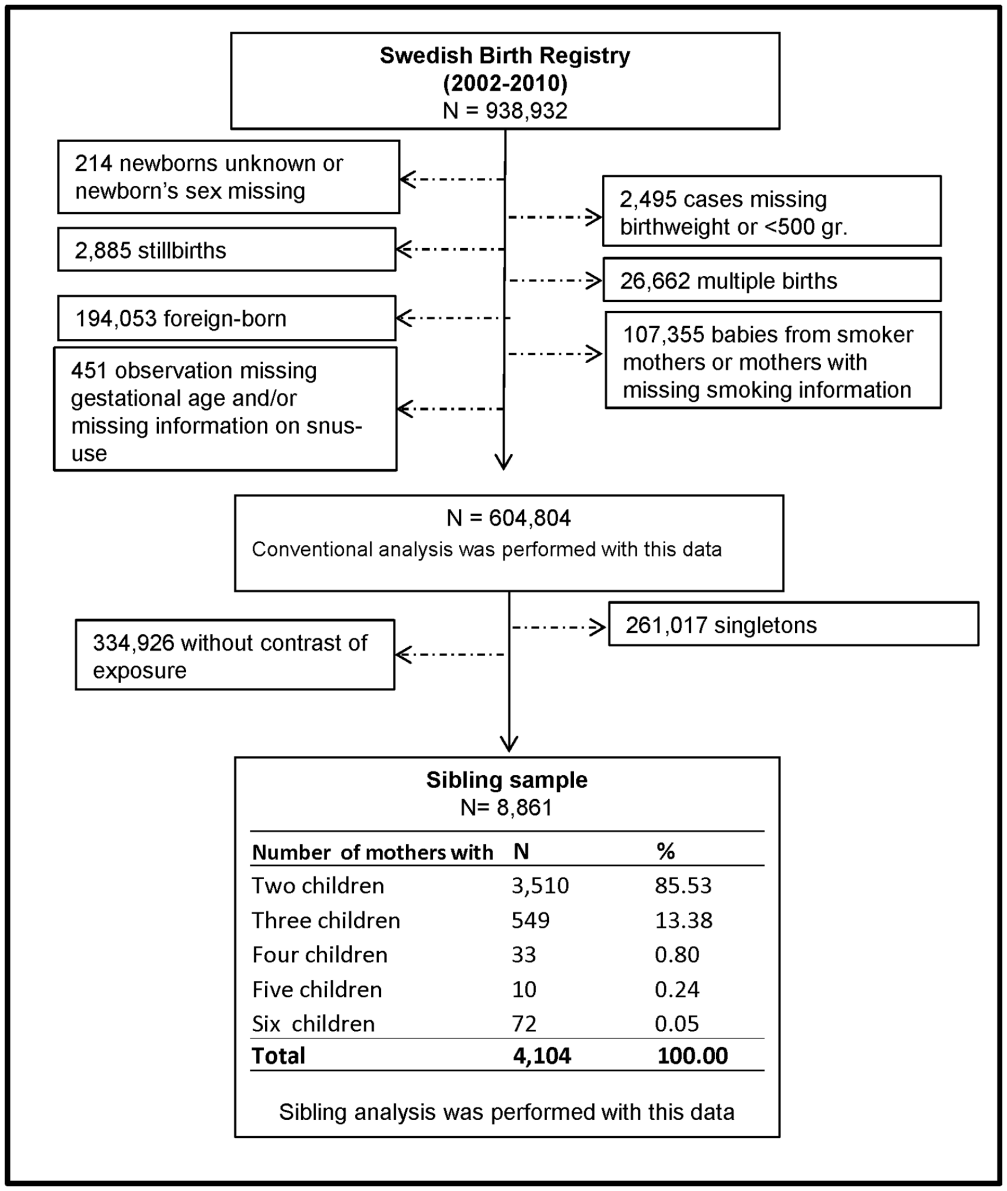
Flow diagram showing the selection of the study population for investigating the effect of maternal snus use during pregnancy on offspring birthweight.

We based our study on the Swedish Medical Birth Register (MBR), which contains approximately 99% of all deliveries occurring in the country [28]. Figure 1 presents the flow diagram with our selection criteria. Of the 938,932 babies born during the period 2002–2010, we excluded babies with unknown or missing sex information (n = 214) and those with missing birthweight or with birthweight less than 500 grams (n = 2,495). To increase the homogeneity of our study population we excluded stillbirths (n = 2,885), multiple births (n = 26,662), because their growth is reduced from 28–30 gestational weeks [29], and babies of foreign-born mothers (n = 194,053). We also excluded mothers with smoking habits or who had missing information on smoking (potential smokers) (n = 107,355), since they might bias the analysis as smoking has a clear effect on birthweight reduction. Because tobacco use has been reported to increase the risk of preterm deliveries [30]–[33] and because a proportion of mothers who have preterm babies do not provide information on smoking in the third trimester, we excluded those cases with missing gestational age or those who, being preterm, have missing information on snus use (n = 451). Since two of the categories of snus use (“missing-no snus use” and “missing-missing”) had very small numbers we also excluded them from the analyses (n = 13). With this final sample (*n* = 604,804) we performed the conventional analysis.

Thereafter, we selected a subsample of siblings with discordant snus-use habits between pregnancies i.e., women who had at least one pregnancy during which they used snus (either in the 1^st^ and/or 3^rd^ trimester) and at least another pregnancy during which they did not use snus in any of the trimesters (reference). This procedure rendered 8,861 discordant siblings and 4,104 mothers. With this sample we performed the corresponding sibling analysis.

### Assessment of Variables

The study outcome variable was birthweight in grams (g). Information on snus use during pregnancy was self-reported and assessed at the first antenatal visit (i.e., between gestational weeks 10 and 12) and in the third trimester (i.e., gestational weeks 30–32) using a questionnaire administered by the midwife. We distinguished between the following: (i) non-users of snus during pregnancy (i.e., those who report using non-use of snus in the first and in the third trimester); (ii) continuous snus use during pregnancy (i.e., those who report using snus in the first and in the third trimester), (iii) quitting snus use (i.e., those who report using snus in the first trimester but non-use in the third trimester); (iv) relapsing snus use (i.e., those who report non-snus use in the first trimester but snus use in the third trimester). We completed this variable including all possible combinations between snus use information and missing data in the two periods (first and third trimester). Thus, we also included (v) “missing”–“snus use” (i.e., those who have “missing” in the first trimester but reported snus use in the third trimester), (vi) “snus use”–“missing” (i.e., those who report snus use in the first trimester and missing in the third trimester), (vii) “missing”–“non-use” (i.e., those with missing snus use in the first trimester and who reported snus use in the third trimester), (viii) “non-snus”–“missing” (i.e., those who reported snus use in the first trimester and missing in the third trimester) and (ix) “missing”– “missing” (i.e., those who did not report information on snus use at any trimester). Categories (vii) and (viii), as we said above, were finally excluded because of the small number of cases.

We adjusted all models for gestational age, birth order, sex, mother’s age, and marital status (see Table 1 for detailed information on the categorization of the variables and the categories used as references in the comparisons).

**Table 1:** Characteristics of the mothers and babies (2002–2010) for the two samples studied.

	Full sample	%	Sibling sample %	
**Mother’s characteristics**				
**Snus use during pregnancy (first-third trimester)**				
Never used snus*	591,690	97.83	4,641	52.38
Continous snus use	2,298	0.38	455	5.13
Quit snus use	4,934	0.82	1,739	19.63
Snus use relapse	1,107	0.18	424	4.79
No use snus -missing	3,313	0.55	1,168	13.18
Snus use -missing	1,462	0.24	434	4.90
**Mothers’ age (years)**				
<20	12,229	2.02	248	2.80
20–24	54,793	9.06	1,117	12.61
25–34*	411,113	67.97	6,087	68.69
35–40	125,972	20.83	1,403	15.83
>40	668	0.11	5	0.06
Missing	29	0.00	1	0.01
**Mothers’ marital status**				
Cohabiting with father*	577,942	95.56	8,427	95.10
Single	5,750	0.95	71	0.80
Other family situation	15,784	2.61	272	3.07
Missing	5,328	0.88	91	1.03
**Newborn’s characteristics**				
**Birth order**				
1 *	275,670	45.58	3,342	37.72
2	230,225	38.07	3,833	43.26
3	75,532	12.49	1,184	13.36
>4	23,377	3.87	502	5.67
**Newborn’s sex**				
Female*	293,890	48.59	4,386	49.50
Male	310,914	51.41	4,475	50.50
**Birthweight mean (n; standard error)**	3599 (604,804; 545)		3647 (8,861; 517)	

* Reference category in the analyses.

### Statistical Analyses

We applied a conventional multiple linear regression analysis to estimate the association between maternal SDP and offspring birthweight for the full sample in order to replicate the common standard procedure, which generally violates the assumption of independence between the observations by including siblings without special treatment. Thereafter, following Iliadou et al’s approach [15], we performed another linear regression analysis to estimate birthweight differences in two subsequent children in a sibling sample that includes both discordant and concordant siblings. Birthweight differences by maternal snus use in two subsequent pregnancies were estimated in both the first and second sibling separately by using linear regression models. Finally, we applied a multilevel linear regression analysis (siblings nested within mothers) [34], [35] using Markov Chain Monte Carlo (MCMC) estimations and orthogonal parameterization [36]. The purpose of this analysis was to obtain mother-specific regression coefficients [37]. By including a random term for the mother, the multilevel regression analysis was adjusted for unknown genetic and maternal environmental factors.

## Results

The socio-demographic and clinical characteristics of the full sample and the subpopulation of siblings are presented in Table 1. Overall, the characteristics of both samples were very similar. However, compared with the full sample, the sibling sample shows a higher proportion of mothers between 20–24 years of age and a lower proportion of mothers in the higher age groups. Furthermore, as expected, the complete dataset has a larger proportion of first-order newborns because this dataset includes children without siblings born in the study period, and these children are excluded from the sibling sample.

Table 2 presents the results of the conventional multiple linear regression analyses. We observed a birthweight reduction for those who used snus throughout the whole pregnancy (i.e., 47 g) those who quit after the first prenatal visit (14 g), and those who relapsed after the first trimester (14 g). The category of “snus use”–“missing” also shows a reduction in 25 g in comparison with mothers who do not use snus during pregnancy.

**Table 2:** Conventional and quasi-experimental sibling analysis of the effect of maternal snus use on offspring birthweight.

	Conventional analysis				Sibling analysis	
Categories of snus use	N (%)	β	CI-95%	N (%)	β	CI-95%
Never used snus (Reference/intercept)	591,690 (97.83)	3581	[3579 3583]	4,641 (52.38)	3615	[3597 3632]
Continous snus use	2,298 (0.38)	−47	[−63 −47]	455 (5.13)	−20	[−52 12]
Quitting snus use	4,934 (0.82)	−6	[−17 4]	1,739 (19.63)	−14	[−31 3]
Snus use relapse	1,107 (0.18)	−4	[−27 19]	424 (4.79)	−14	[−46 18]
No snus use-missing	3,313 (0.55)	33	[20 47]	1,168 (13.18)	4	[−16 24]
Snus use-missing	1,462 (0.24)	−25	[−45 −5]	434 (4.9)	−33	[−66 −0.08]

All models were adjusted for gestational age, marital status, maternal age, birth order, sex of the newborn.

There were no statistically significant differences in the quasi-experimental sibling analysis when comparing any amount of snus use with no snus use in any of the categories but in the category “snus use”–“missing” (33 g.). Similar results are found when using the exposure variable as dichotomy (never use snus/any snus use) so the uncertainty does not depend on the disaggregation of the snus use exposure (data not shown in tables). Compared to never used snus (reference), mothers who use snus in any trimester have lighter babies on average (−19; 95%CI −27; −11) based on the conventional analysis while no statistical differences are observed with the sibling analysis −12; 95%CI −25; 2).

Table 3 presents the results from comparing birthweight differences in the subsequent study pregnancy in relation to snus use habit. Only mothers who use snus in the two subsequent pregnancies show a statistically significant reduction on birthweight and, this reduction, is more intense in the second study pregnancy (56 g.) than in the first one (41 g.).

**Table 3:** Maternal snus use and birthweight differences based on 144,017 mothers with two subsequent births.

Snus use during pregnancy		1^st^ study pregnancy			2^nd^ study pregnancy		
1^st^	2^nd^	N	Mean Birthweight (SD)	Adjusted Birthweight (95% CI)	N	MeanBirthweight(SD)	Adjusted Birthweight (95% CI)
No	No	140,761	3608 (534)	Reference	140,761	3609 (532)	Reference
Yes	No	1,277	3628 (522)	4 [−21 30]	1,277	3630 (499)	12 [−14 37]
No	Yes	1,253	3617 (520)	−9 [−35 16]	1,253	3629 (558)	23 [−2 49]
Yes	Yes	726	3580 (547)	−41 [−74 −7]	726	3572 (527)	−56 [−90 −22]

All models were adjusted for gestational age, marital status, maternal age, birth order, sex of the newborn.

## Discussion

The conventional linear regression analysis suggested that using snus during pregnancy was associated with a slight reduction in offspring birthweight (47 g), which appears to confirm the results from a previous observational analysis [19]. However, the results obtained with the quasi-experimental sibling analysis showed that snus exposure during pregnancy reduces birthweight although, this reduction, is minor (20 g) and not statistically significant. The sample of siblings with contrast of exposure made it possible to reduce unaccounted confounding, but this selection also reduces the number of studied subjects and, thereby, the uncertainty of the estimates increases. However, our analysis strongly suggests that snus has a minor effect on birthweight reduction and, therefore, that nicotine does not seem to be the main mechanism involved in the association between smoking during pregnancy and birthweight. This result is in line with a previous conventional analysis that did not find an association between nicotine replacement therapy (i.e., patch, gum, inhaler), or other types of smokeless tobacco (i.e., iqmik, commercial chew) and offspring birthweight, [16], [38].

Our findings should not be interpreted as suggesting that the use of snus is a healthier alternative to smoking during pregnancy because snus also has harmful health consequences [39], [40] and it has been found to be addictive [26], [41]. In fact, previous perinatal studies have found that snus is associated with stillbirth, preeclampsia, and very preterm and preterm babies [19], [32], [42], although the causal strength of those observations remains to be confirmed. Our study contributes to the ongoing debate [26], [27], [43] on the health implications of using snus.

Our results, moreover, are of particular importance to identifying the mechanisms through which tobacco consumption may influence birthweight because this knowledge is necessary for planning effective therapeutic interventions [44]. Using a quasi-experimental sibling analysis, our study supports the hypothesis that the adverse effect of smoking during pregnancy in birthweight reduction is not mediated by nicotine but rather related to toxic products from the tobacco combustion or other smoking constituents. Cigarette smoke contains not only carbon monoxide, a well-known poisonous product of combustion [45], but also such chemical substances as carcinogens, toxic heavy metals, and many other elements of untested toxicity [4]. Moreover, cigarette smoke facilitates nicotine absorption when the smoke reaches the small airways and alveoli of the lung, since only small amounts of nicotine are absorbed through the buccal mucosa [7]. In other words, smoking includes toxic mechanisms that are absent in smokeless tobacco.

In addition to the quasi-experimental approach, the current study has a number of other strengths. Our analyses are based on a nationwide birth registry covering nearly 99% of the deliveries occurring in Sweden [28]. This dataset contains information on snus use at two moments during pregnancy (first and third trimester). This allowed us to identify siblings discordant in exposure to snus, and to distinguish between mothers with different patterns of snus use. Moreover, we were able to control for variables which vary between pregnancies (such as gestational age, birth order and maternal age). These adjustments are essential since the strength of the sibling analyses is based on constant environmental factors and genetic background [46], [47].

However, our investigation also has several limitations. The self-reported information on snus use by the mothers may be inaccurate, which may lead to information bias towards the null. For instance, if a true snus user only reported using snus in one pregnancy but not in the other (creating false contrast of exposure), the association between maternal snus use and birthweight will become attenuated [48]. Nevertheless, a study performed in Sweden comparing self-reported nicotine exposure and plasma levels of cotinine in early and late pregnancy concluded that self-reported information on nicotine exposure had acceptable validity [49]. Although this study did not validate self-reported information on snus, the societal attitude against snus is less severe than that against smoking, which might promote more accurate self-reporting of snus habits than of smoking habits. Moreover, the fact that we had information on snus use during two moments during pregnancy may increase our probability of identifying true snus users than if we only had one observation.

Additionally, we used a sibling analysis to account for unmeasured maternal confounders, but other temporal confounders may exist that were not considered. Moreover, the siblings were only matched by their mothers. At the time of our investigation, we did not have information about the father and, therefore, some of the siblings may have only been half siblings. Moreover, it is possible that the association between SDP and birthweight is confounded by smoking in the father, influencing the child through passive smoking in the mother or smoke exposure among those who do not smoke or quit.

In spite of the fact that we use a population register that contains all deliveries in Sweden for a period of eight years, we were not able to study other outcomes derived from birthweight such as low birthweight (<2,500 g) or small-for-gestational-age, the last of which has been recently explored through a conventional analysis [50], because the sibling analysis for dichotomous outcomes restricts the sample considerably and, therefore, it does not allow us to reach any conclusion.

In summary, applying a quasi-experimental sibling design to a large database with detailed information on tobacco use, we observed that snus exposure during pregnancy has a minor effect on birthweight reduction. More empirical evidence is required to confirm this result, especially with a larger sibling sample. Nevertheless, our results suggest that the adverse effect of smoking during pregnancy on offspring birthweight may be explained by the combustion or other products of smoking rather than by nicotine.
